# Vital Role of In-House 3D Lab to Create Unprecedented Solutions for Challenges in Spinal Surgery, Practical Guidelines and Clinical Case Series

**DOI:** 10.3390/jpm12030395

**Published:** 2022-03-04

**Authors:** Koen Willemsen, Joëll Magré, Jeroen Mol, Herke Jan Noordmans, Harrie Weinans, Edsko E. G. Hekman, Moyo C. Kruyt

**Affiliations:** 1 Department of Orthopedics, University Medical Center Utrecht, 3584 CX Utrecht, The Netherlands; j.magre-2@umcutrecht.nl (J.M.); jjh.mol@hotmail.com (J.M.); h.h.weinans@umcutrecht.nl (H.W.); m.c.kruyt@umcutrecht.nl (M.C.K.); 2 3D Lab, Division of Surgical Specialties, University Medical Center Utrecht, 3584 CX Utrecht, The Netherlands; 3 Department of Medical Technology and Clinical Physics, University Medical Center Utrecht, 3584 CX Utrecht, The Netherlands; h.j.noordmans@umcutrecht.nl; 4 Department Biomechanical Engineering, Delft University of Technology, 2628 CD Delft, The Netherlands; 5 Department of Biomechanical Engineering, Twente University, 7522 NB Enschede, The Netherlands; e.e.g.hekman@utwente.nl

**Keywords:** spinal implants, pedicle guides, implants, 3D-printed, biomechanical

## Abstract

For decades, the advantages of rapid prototyping for clinical use have been recognized. However, demonstrations of potential solutions to treat spinal problems that cannot be solved otherwise are scarce. In this paper, we describe the development, regulatory process, and clinical application of two types of patient specific 3D-printed devices that were developed at an in-house 3D point-of-care facility. This 3D lab made it possible to elegantly treat patients with spinal problems that could not have been treated in a conventional manner. The first device, applied in three patients, is a printed nylon drill guide, with such accuracy that it can be used for insertion of cervical pedicle screws in very young children, which has been applied even in semi-acute settings. The other is a 3D-printed titanium spinal column prosthesis that was used to treat progressive and severe deformities due to lysis of the anterior column in three patients. The unique opportunity to control size, shape, and material characteristics allowed a relatively easy solution for these patients, who were developing paraplegia. In this paper, we discuss the pathway toward the design and final application, including technical file creation for dossier building and challenges within a point-of-care lab.

## 1. Introduction

Additive manufacturing, commonly known as 3D printing, has been adopted as one of the key elements of future medical care. The entire process has evolved and progressed over the last 20 years, first in laboratories for fundamental research and subsequently for actual clinical care [1,2,3]. New tools to treat complex and previously untreatable surgical problems while increasing accuracy are provided 3D technology [4]. However, to optimally benefit from such front-running technological enhancement, close interaction between all stakeholders and especially between technical and medical personnel is mandatory [3]. Therefore, an increasing number of hospitals are establishing a 3D printing point-of-care facility in which the opportunities of 3D printing, in terms of technical possibilities and legal/regulatory challenges, can be fully explored [5,6]. Such a facility, frequently known as a (point-of-care) 3D lab, uses the output of established state-of-the-art clinical image modalities such as the newest CT and MRI and subsequently post-processes the data into digital anatomical models to better embody the patient and to allow interaction with surgeons for the development of custom-made medical devices [7,8,9,10]. To enable this, the 3D lab personnel act as a multidisciplinary team to remove boundaries such as jargon, and should be able to quickly produce 3D models, prototypes, and even implants under governance of an appropriate quality management system (ISO 13485:2016) [5].

This 3D technology is especially important for tertiary referral hospitals, which primarily function as a specialized center for complex cases and ultimately as a safety net for last-resort cases [6,11]. These hospitals have an academic setting where research, innovation and unique treatments come together [11]. As a result, exceptional cases that demand exceptional solutions are referred to these hospitals. To optimally serve in this academic role, medical doctors, engineers, and researchers work closely together in fundamental and innovative research projects within the 3D lab [3]. 

In this paper, we demonstrate two additive manufacturing pathways that were created as a collaboration between a tertiary spinal surgery unit and an established point-of-care 3D lab within one academic hospital. The first pathway is a patient-specific device to guide cervical pedicle screws in very young children or patients with extraordinary spinal anatomy. The second pathway is an additively manufactured implant that is used as a spinal prosthesis to bypass the severely distorted and mechanically unstable spinal column. For both pathways, background information is given, followed by the methodology and clinical results.

## 2. Pathway

### 2.1. Click-on Guide for Cervical Pedicle Screws the Second Level Heading

#### Background

**Figure 1:**
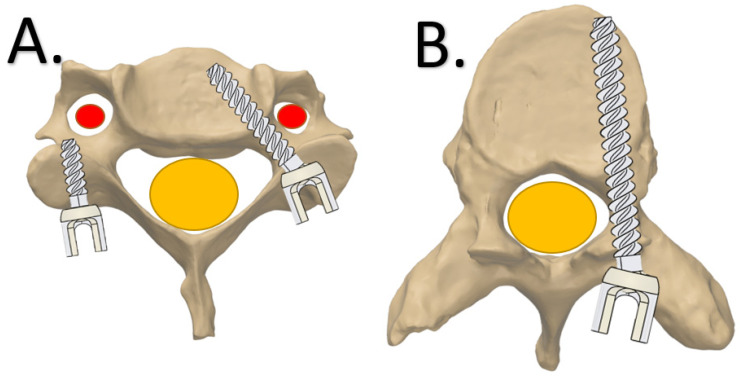
Screw placement in vertebrae, axial view. (**A**) Cervical vertebrae with a lateral mass screw (**left**) and pedicle screw (**right**). (**B**) Thoracic vertebrae with a right-sided pedicle screw. The red area is the cervical artery and the yellow area is the spinal cord.

For spinal surgery, pedicle screws that run from posterior to anterior are currently the most used fixation technique. Since the groundbreaking work of Suk et al. in 1995 [12], spine surgeons also adopted its use for challenging locations such as the scoliotic thoracic spine [13,14]. Although the pedicle screw trajectory seems dangerous, as it passes immediately next to the spinal canal (Figure 1), its use has been demonstrated to be safe in experienced hands, even when the screws are not positioned perfectly. About 10% of the free-hand-positioned screws show some breaching of the pedicle, medially or laterally, without clinical consequences [15]. For the cervical spine, mispositioned pedicle screws are less forgiving as the vertebral artery constitutes the lateral border of the trajectory, which makes a “lateral breaching” intolerable (Figure 1). For that reason, most surgeons prefer the weaker lateral mass screws for cervical spine fixation [16]. In young children, the lateral mass has not yet developed and cannot serve as a foundation for screws, especially if distraction or pull-out forces are expected [16]. Alternative options such as hooks and wires, can be used but have serious risk of neurological complications. Consequently, placement of cervical pedicle screws to obtain strong cervical spine foundations is currently a high-risk procedure in very young children [17,18]. Even 3D navigation, which can be used for this application in adults, may not allow sufficient accuracy due to the movement introduced by mechanical ventilation and the limited intraoperative image resolution [17,18,19].

**Figure 2:**
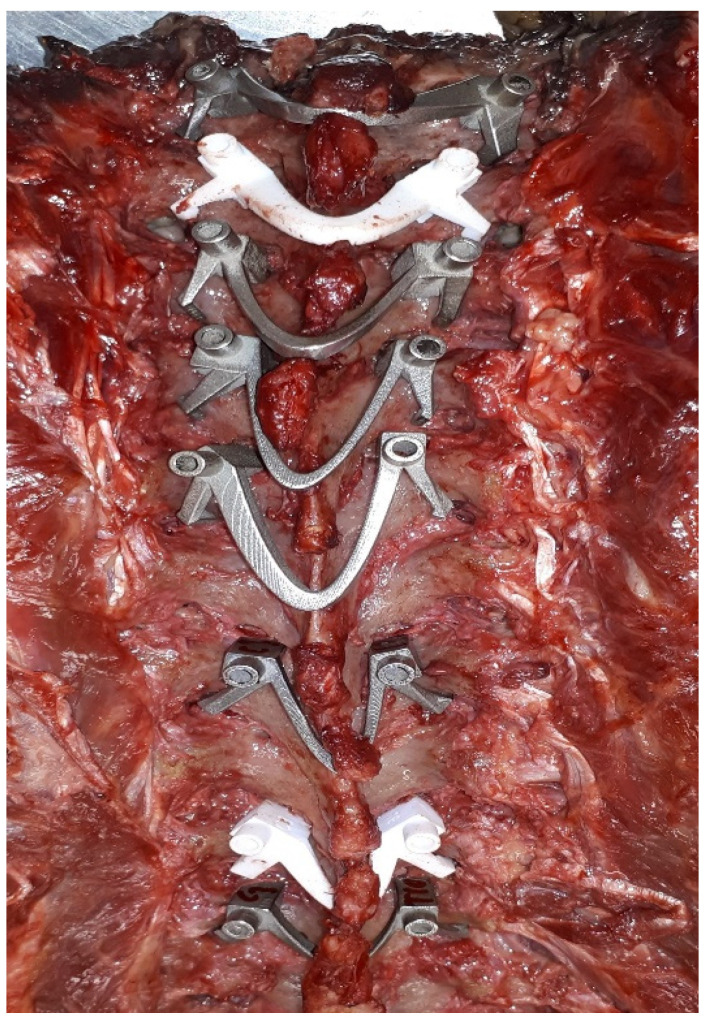
Examples of metal and polymer lay-on spine guides in thoracic cadaveric spine.

To aid surgeons with pedicle screw positioning in adults, patient specific 3D-printed drill guides have been developed by several groups [16,20,21,22]. These drill guides are based on direct surface contact on the laminae and transverse and spinous processes of one or sequential vertebrae, but this does not always reach the required accuracy for cervical use [23,24,25,26]. In both metal and polyamide lay-on drill guides, screw tip deviation can still be >2 mm (Figure 2) [23,24,25,26], which is unacceptable in the axial plane of cervical vertebrae, especially in young and small children [17,18]. 

**Figure 3:**
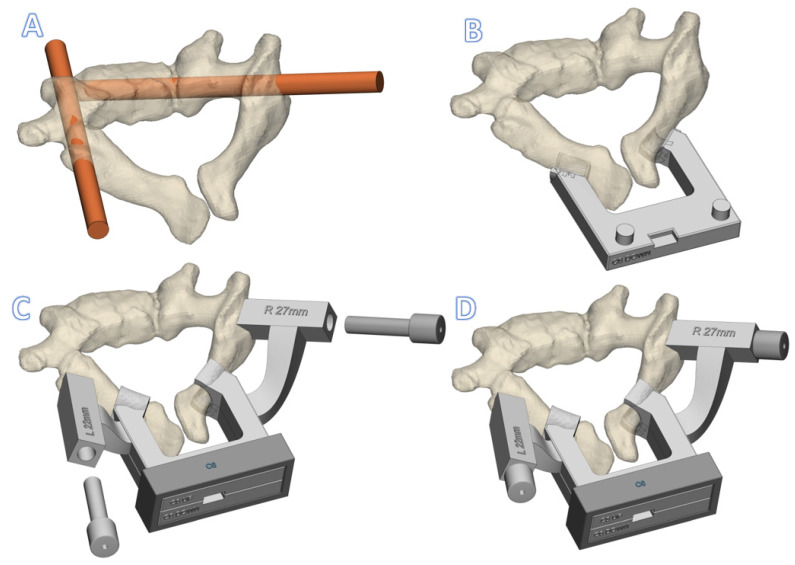
Rendering of a C6 click-on spine guide (**A**). The preferred screw trajectories (**B**). The interconnected lamina hooks of the caudal part (**C**). The cranial part including the drill guide cavities and the (metallic) drill guides (**D**). Fixation of the assembly with the fixation box, included the inserted drill guides.

Moreover, the surface of the lateral apical processes, needed for optimal rotational stability, is difficult to clean from soft tissue, especially in the pediatric cervical spine where apophyseal cartilage is present. This soft tissue component hampers a perfect fit between the lay-on guide and vertebra. Additionally, the spinous process needed for sagittal stability cannot be used in the pediatric cervical spine. The only and most accessible smooth bone surfaces of the pediatric cervical spine are found at the laminae. However, the more medial laminae do not allow sufficient stabilization of the lay-on guide. Therefore, since the laminae is oval in cross-section, a clamp that is placed around it will effectively block all degrees of freedom except perpendicular to the sagittal plane. This motion can be blocked with extensions that rests on the entry point of the pedicles. Consequently, we designed a click-on assembly that can be clamped around the bilateral laminae and harbors a drill guide in the upper part (Figure 3).

## 3. Device Description of Click-on Pedicle Guides

### 3.1. Anatomical Data Acquisition

First, a CT scan is made of the patient with ≤1 mm slice thickness (250 mAs, 120 kV). We consider this resolution sufficient and in accordance with the “as low as reasonably possible” principle for radiation. Thereafter, the DICOM data are transferred to medical licensed segmentation software (Mimics, Materialise NV, Leuven, Belgium) to create an anatomical model of the spine of the patient. Together with the surgeon, the screw trajectory is planned for the desired vertebrae. Thereafter, the anatomical models and screw trajectories are exported to medical certified CAD-CAM software (3-matic, Materialise NV, Leuven, Belgium) to design the click-on pedicle guides.

### 3.2. Design 

The patient-specific cervical pedicle click-on guide consists of three components that are assembled on the laminae during the surgery. First, the lower part contains two lamina hooks that are designed to exactly fit the caudal side of the laminae. Then, the top part is designed, which has two laminar hooks that exactly fit on the cranial side. Moreover, the top part also contains two extensions with cylindrical cavities for metallic drill guides. The exact trajectories of those drill guides are established in collaboration with the surgeon. Thereafter, the lower and top parts dock and can be rigidly fixed to each other using a simple fixation box (Figure 3). Finally, cylindrical metal drill guides are inserted in the guide cavities. More details on the design are provided in the Figures S1–S3.

### 3.3. Guide Production

All three parts of the guide were 3D-printed under the ISO13485 quality management system. The guides were produced using selective laser sintering of nylon powder (PA12) with a printing accuracy of 0.12 mm in all directions (P110, EOS, Krailing, Germany). Before sterilization, the accuracy of the assembly was checked on the receiving cervical vertebrae that were printed separately with the same resolution. Thereafter, the guides were sterilized at our in-house sterilization facility by manual cleaning and standard autoclave sterilization (ISO 17665–1:2016 and EN 285) and sterile packaging (ISO 11607–1:2019).

### 3.4. Preclinical Tests

**Figure 4:**
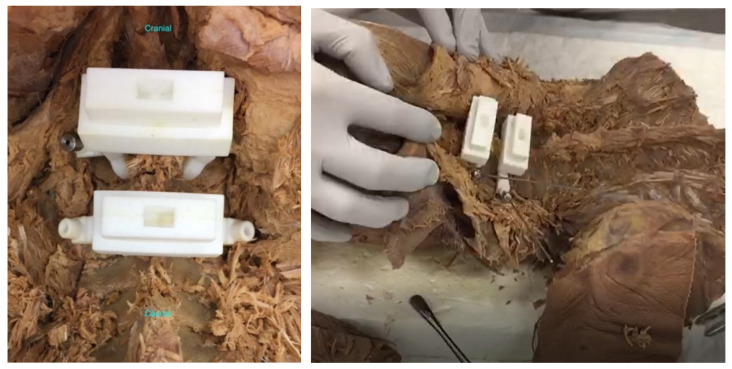
Photos of the cadaveric experimental set-up to test the accuracy of the click-on pedicle guides.

The nylon versions of the click-on spine guides showed excellent stability and acceptable accuracy in cadaveric tests (Figure 4). The average entry point deviation was 0.9 8 ± 0.38 mm and average angular deviation from the midline was 1.75° ± 0.62° (*n* = 4).

## 4. Clinical Application

The cervical guide has been applied in two cases of spinal distraction and one case of emergency treatment. In our center, we use spring distraction as a growth guidance technique for severe deformities that cannot be controlled with less invasive techniques such as braces or halo vests [27]. For cervicothoracic congenital deformities especially, the technique has shown the potential to not only control but even to reduce the deformity [28]. To allow continuous distraction forces on the cervical vertebrae, a strong and reliable foundation is a prerequisite. Two patients (age 4 years) have currently been treated with spring distraction delivered to cervical pedicle anchors and, in both cases, a click-on spine guide was designed and used intraoperatively.

**Figure 5:**
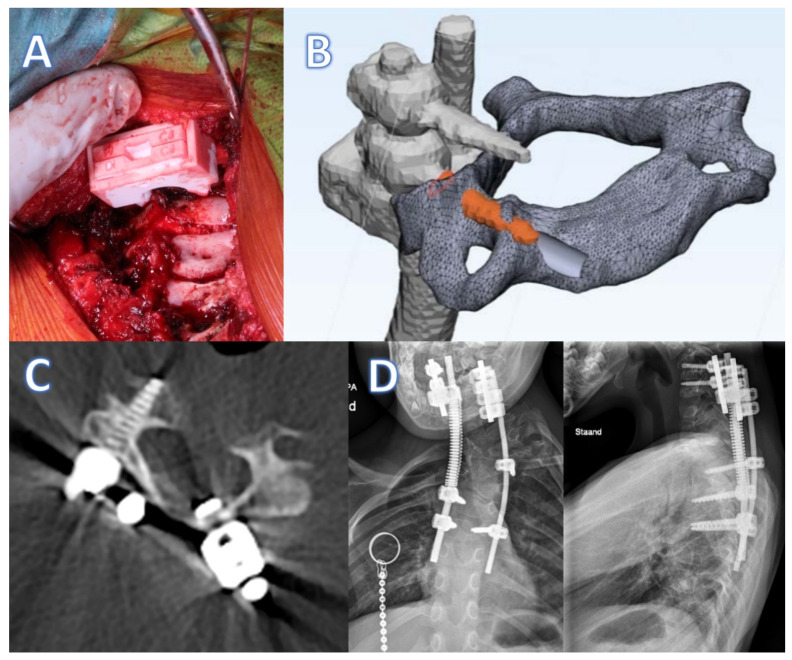
Click-on guide case 1. (**A**) Intraoperative use of the click-on guide, exposure of the operative site limited due to interference with the posterior skull. (**B**) Slight upward deviation of the C6 screw (orange) in comparison to the planned trajectory (gray). (**C**) Post-operative axial CT reconstruction with the screw precisely through the pedicle. (**D**) Post-operative radiographs of the instrumented spine.

The first case was a 4-year-old boy with congenital scoliosis. For safety reasons, the C5 and C6 pedicle screws were only inserted unilaterally at the distraction side. Intraoperatively, after insertion of the K-wire, the position was checked with radiographs, after which 3.5 mm pedicle screws were placed. The procedure went well; however, on the postoperative CT, we noticed a slight cranial deviation in the sagittal plane. This was likely caused by forces on the device due to insufficient exposure proximally, something that we took care of in the later procedures. There were no medial or lateral breaches and the distraction force (of 75N) was well-sustained. This case has been followed up for almost 3 years now (Figure 5).

**Figure 6:**
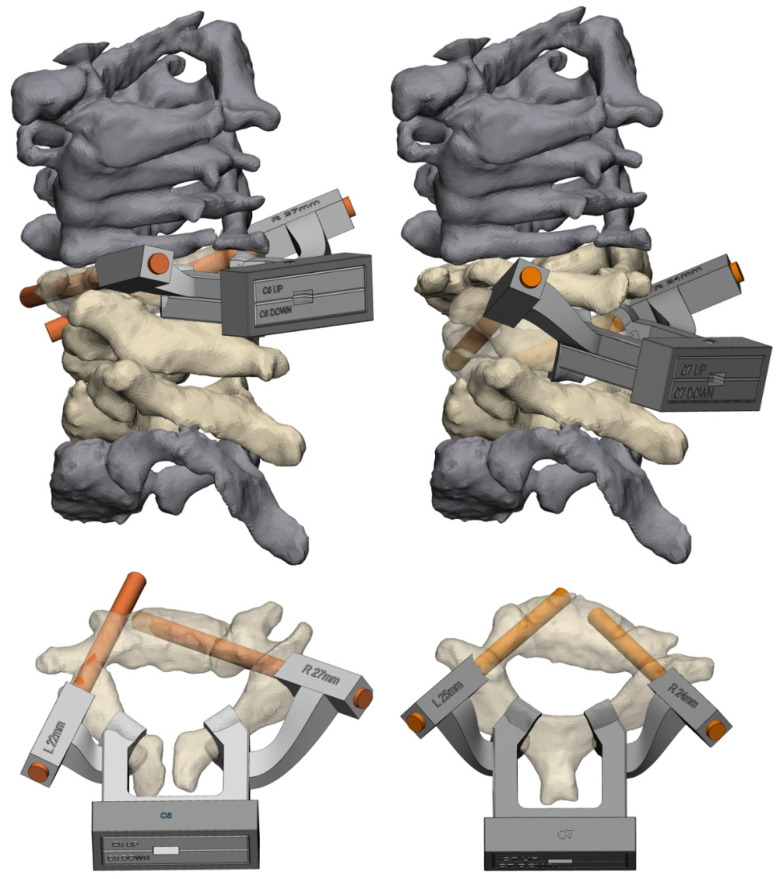
Click-on guide case 2. On the left side, the guide for C6; on the right side, C7. The orange cylinders are the target screw trajectories in the design.

**Figure 7:**
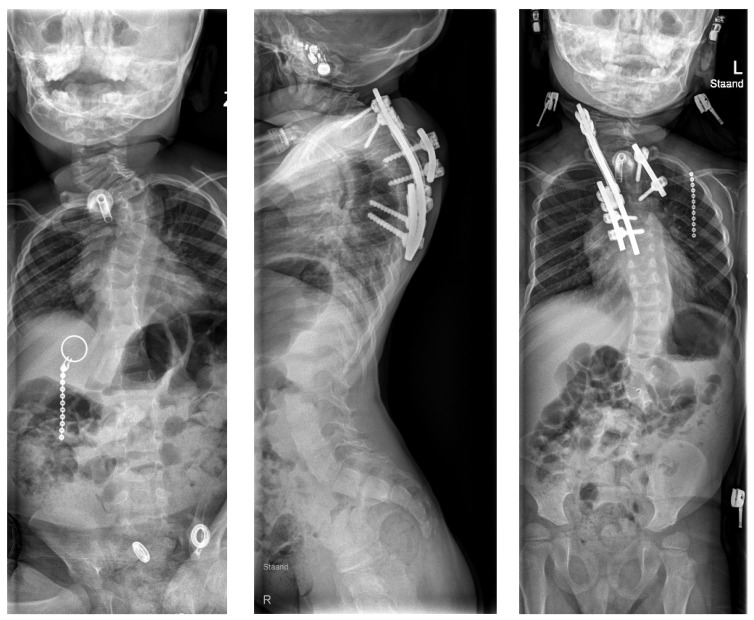
Case 2: preoperative AP sitting radiograph (**left**) and postoperative lateral (**middle**) and AP (**right**) standing radiographs.

The second case was a 4-year-old boy with Pierre Robin syndrome and severe high thoracic scoliosis. A halo vest had failed to control the curve; therefore, the spring distraction system was considered the best treatment. Unilateral placement of C6 and C7 pedicle screws was uneventful and smooth (<10 min per screw) with use of the click-on guides (Figure 6). The latest follow-up is 9 months (Figure 7).

**Figure 8:**
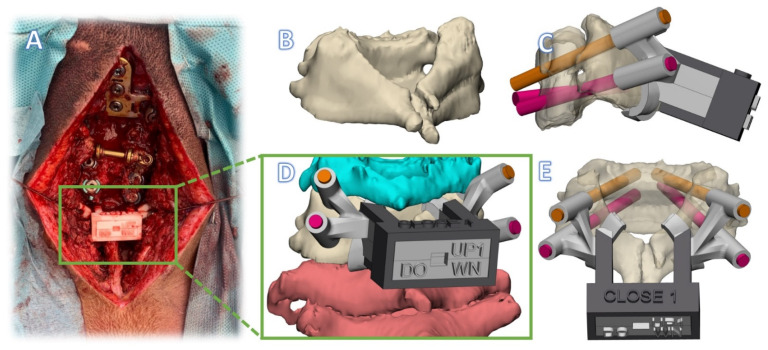
Click-on guide case 3. (**A**) Intraoperative photo. (**B**) Fused vertebrae C5-C6. (**C**) Lateral view of alternative guide design with four pedicle guides from two levels in click-on guide system. (**D**) Rendering of guide with cranial and caudal vertebrae also visible. (**E**) Posterior view of the guide system.

The third case did not receive a spring distraction; it had a more urgent situation. This 8-year-old girl presented in the emergency setting for basilar impression and impeding paraplegia due to high cervical congenital anomalies. Decompression of the C1 lamina was needed and, due to the absence of C2 pedicles, an occiput to C3 fusion was performed. We mistakenly used only lateral mass screws (6 mm), which could not prevent recurrence of kyphosis and signs of paraplegia within 3 weeks. Therefore, immediate halo traction was provided, which reduced the head and reversed the paraplegia. Because this was not a permanent solution, we decided to revise the internal fixation with C5–6 pedicle screws and a free-hand C2-to-C1 fixation screw. Due to the available template for the click-on guides and especially the in-house availability of the 3D laboratory, the guide was produced within 1 week, which allowed for successful semiacute revision. In this case, there was a fusion of certain vertebrae, which resulted in a combined guide for two levels of vertebrae (C5–6) within one system (Figure 8). Currently at 9 months follow-up, the patient is fully recovered and does not show signs of fixation failure.

## 5. Pathway 2: Spinal Column Prosthesis

### Background

**Figure 9:**
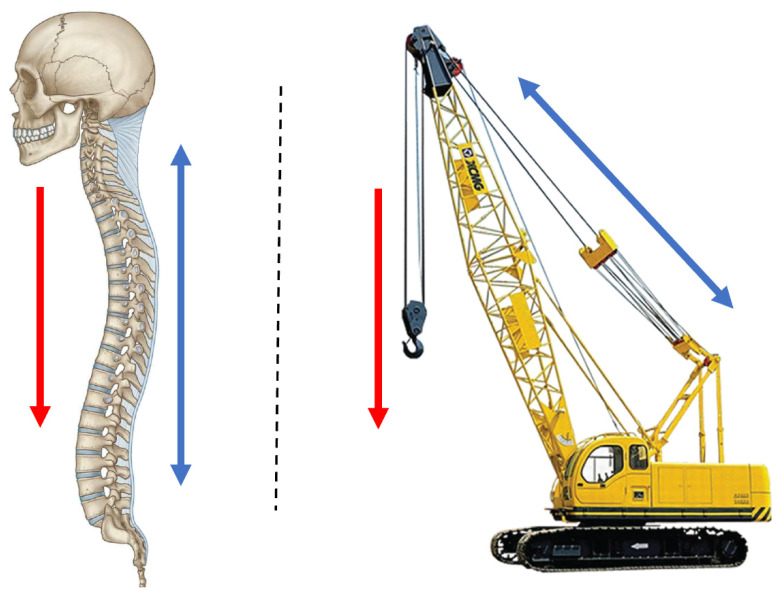
Resemblance of spinal balance equilibrium with a crane. Red arrows represent (body) weight carried by the vertebrae (**left**) and crane arm (**right**). The blue arrows represent the stabilizing counterforce (tension) by the posterior ligamentous complex (**left**) and tension cables (**right**).

The spinal column has been described as a mechanical construction that resembles a crane (Figure 9). The metaphor of a crane is especially helpful to determine the cause of instability after spinal trauma. The key elements of a stable construction are the anterior column to support axial compressive forces and the posterior ligamentous complex that functions as a tether [29,30]. As long as the posterior tether mechanism and facet joints are intact, kyphosis is prevented even when individual vertebral bodies collapse. However, when anterior support fails, for instance, due to a lytic disease involving several vertebrae and/or the facet joints, the posterior tether cannot stabilize the construction and progressive kyphosis results, often inducing paraplegia. Classic examples of this are lytic metastases, tuberculosis and neurofibromatosis. Additionally, neurofibromatosis causes severe scoliosis, which leads to a complex 3D deformity. When kyphosis occurs in such a complex deformity, it extends over several vertebrae and involves the cervicothoracic region. In this case, restoration of anterior support with fibular or rib grafts is extremely difficult, very invasive, and has a high chance of complications [31]. This is because internal chest structures such as the heart and bronchi do not allow a bulky implant, and incorporation of the graft bone over a long distance is hampered.

To circumvent these difficulties, we developed a 3D-printed personalized spinal prosthesis. The custom-made implant can be inserted in a way that is minimally invasive and allows for the incorporation of bone into the porous ends of the implant. It has a massive titanium stem that exactly follows the spinal contour with a substantial cross-sectional surface to prevent fatigue failure in time [3].

## 6. Device Description of Spinal Prosthesis

### 6.1. Anatomical Data Acquisition

First, a CT scan is made of the patient with ≤1 mm slice thickness (250 mAs, 120 kV). Thereafter, the DICOM data are transferred to medical licensed segmentation software (Mimics, version 24.0, Materialise NV, Leuven, Belgium) to create an anatomical model of the spine of the patient. Together with the surgeon, the appropriate vertebrae above and below the scoliotic segment are selected, after which the anatomical model containing the selected vertebrae are exported to medical certified CAD-CAM software (3-matic, version 15.0, Materialise NV, Leuven, Belgium) to design the bridging spine implant.

### 6.2. Design

**Figure 10:**
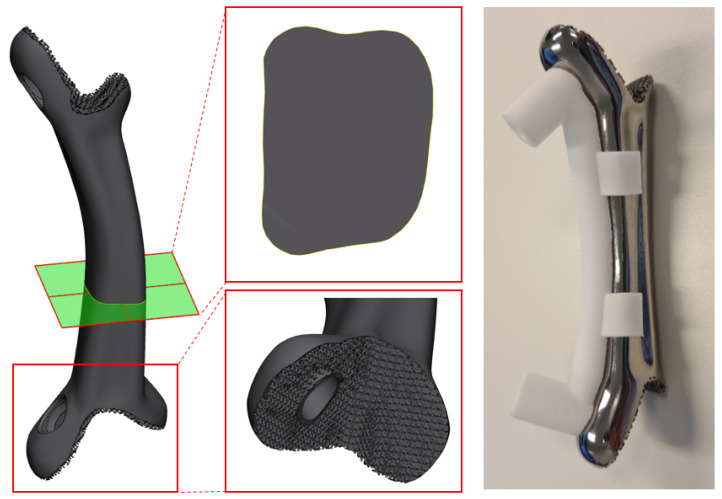
The implant designs. **Left** A rendered model of the implant. **middle** Cross-section (**top**) and a close-up of the porous interface (**bottom**). **right** The actual 3D-printed implant with a nylon drill guide attached.

The biomechanical spinal strut or bridge prosthesis consists of two docking parts with a porous interface (Figure 10 (left)) and a solid bridging part (Figure 10 (right)). A more detailed design description of the prosthesis is provided in the Figure S4. As part of the recommended dossier building for any medical device, a risk analysis was performed by a multidisciplinary team. 

#### Implant Production

The implants were 3D-printed using medical-grade titanium alloy (Ti6Al4V ELI grade 23). The printing was performed with a direct-metal-printing 3D printer DMP320 (3D Systems, Leuven, Belgium). Postprocessing included hot isostatic pressing treatment, mirror polishing, ultrasonic cleaning, and quality control. Final (manual) cleaning and standard autoclave sterilization of the implants was performed in-house. All accompanying drill guides, dummies, and trial implants were produced from nylon PA12 following the same production steps as the click-on pedicle screw guides.

### 6.3. Clinical Experience with Spinal Prosthesis

The spinal bridge prosthesis has been applied in three cases with spinal instability and posterior rod failure. The first two patients of this pathway were described previously in a paper on regulatory issues involved in developing 3D printed implants [3]. The first patient was a 16-year-old male suffering from neurofibromatosis type 1 and recurrence of kyphosis despite previous attempts of posterior fusion. He presented with paraplegia for which he was treated with halo gravity traction and revision of posterior instrumentation. Since this would definitely fail in time, we searched for a possibility to provide anterior support from C6 to T11. The main difficulties were the complicated 3D anatomy including the position of the vena cava and right bronchus and the absence of a bone bed. The 3D prosthesis that we made, after a 6 month design process that went back and forth between designer and surgeon, could be inserted within 2 h via two separate incisions and had an excellent fit [3]. Follow-up is three years now without signs of failure. 

The second case was a 69-year-old woman with vanishing bone disease of the lower cervical spine. She had several treatments for vertebral stability within the last 20 years, but the posterior instrumentation repeatedly failed and she presented with severe deformation and paraplegia. Fortunately, paraplegia recovered after halo traction and the spine was temporarily stabilized with posterior instrumentation and a halo vest. Using the procedural template of the first patient concerning design as well as regulatory, safety, and dossier implementation, we could produce a prosthesis within 6 weeks. This prosthesis provided support from C5 to T2 and was inserted with an extended anterolateral cervical approach [3]. This case has been followed up for 2.5 years now. 

**Figure 11:**
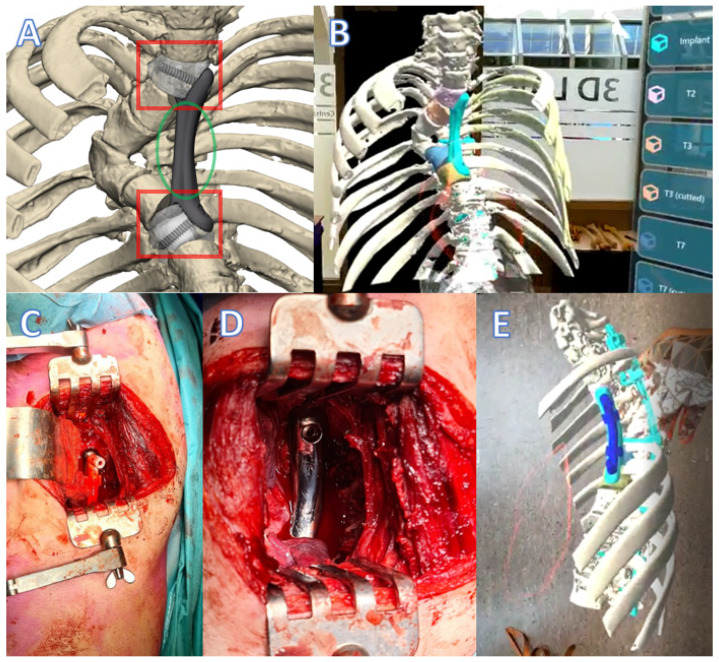
Spinal column prosthesis case 3. (**A**) Design of the spinal column prosthesis. Within the red squares are the partially porous bone connectors. In the green circle the bridging part of the implant is visible. (**B**) A still from a recording of a preoperative HoloLens surgical training. (**C**) Intraoperative photo of the prosthesis with a drill guide in situ (left anterior, right posterior). (**D**) close-up after screw fixation. (**E**) A still from a recording of the preoperative HoloLens surgical training (same approach as in (**D**)).

**Figure 12:**
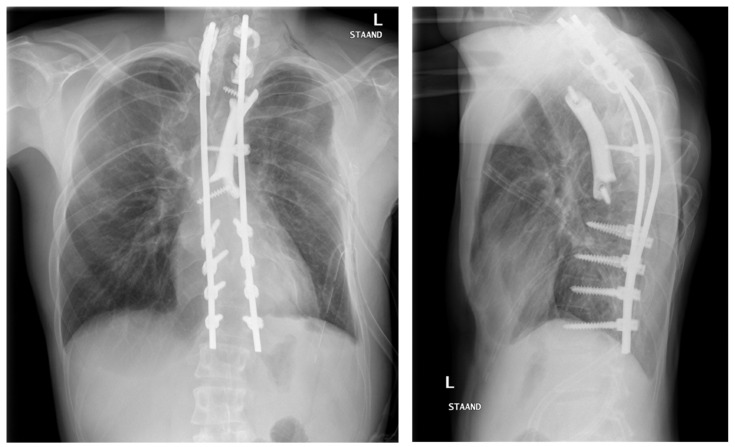
Case 3: Anterior-posterior (**left**) and sagittal (**right**) radiographs postoperation.

The third patient was referred in time, before paraplegia had occurred. This 20-year-old man had a posterior stabilization of his NF1 associated dystrophic kyphoscoliosis (similar to the first patient) five years earlier, but the rods fractured and the kyphosis increased due to absence of anterior support. This timely referral made the procedure much easier, especially for the patient, as halo traction was not required and a two-stage treatment could be planned. With the revision of the posterior system, we deliberately inserted a screw that protruded anteriorly to serve as a reference for the anterior prosthesis that would be placed in the second stage. Due to the nature of the deformity, the prosthesis had to be inserted posterior to the heart on the left side, extending from T2 to T8 (Figure 11 and Figure 12). Before actual surgery, the exposure and order of events of the procedure were simulated in the 3D lab using a HoloLens (Microsoft, Redmond, Washington, DC, USA). We used a posterolateral approach with partial resection of rib 3, 4, and 5. The three-hour procedure happened without issues and resulted in a tight fit of the implant. At 3 months follow-up the implant was stable, and no material breakage occurred. Clinical follow-up is 6 months now.

## 7. Discussion

To treat exceptional spinal cases, exceptional solutions are needed. In this paper, we described the application of patient-specific 3D-printed devices for such solutions and strived to emphasize the potential of this technology, especially when available at an in-house 3D lab. To optimally explore the opportunities of additive manufacturing and 3D personalized design technology in terms of unrecognized possibilities and unmet needs, medical doctors, engineers, and researchers are working closely together in fundamental and innovative research projects.

Medical technology is a fast-growing sector with new devices entering the market each day [32,33]. To protect patients from poorly designed or not sufficiently tested medical devices, new legislation was introduced in 2021.The European Medical Device Regulations (MDRs) describe the precise legislation regarding the development of all medical devices [34]. Moreover, in the MDRs, there is a special section (Annex 13) for the production of patient-specific medical devices, which is important as the growing additive manufacturing industry has enabled an increasing number of hospitals to produce their own in-house-designed products [34]. The MDR dictates that hospitals that produce such patients-specific devices are now also the legal manufacturer of these devices, making them responsible for their quality. This includes the devices presented in this study, which were developed to treat rare cases. To mitigate risks and comply with this new legislation, all in-house development of medical devices needs an appropriate Quality Management System (ISO13485) and multidisciplinary expertise for dossier building. Thankfully, we anticipated this change and started early with a multidisciplinary collaboration for 3D technology within our hospital and with affiliated technical universities [5]. This collaboration resulted in the current 3D lab with medically trained staff and engineers and had support of the Medical Technology and Clinical Physics department that was already ISO-13485-accredited for medical device development. Factors that need to be considered according to the ISO standard include risk analysis, traceability of procedures and implants, ISO certifications of critical suppliers, and a technical rationale per medical device.

Nowadays, our 3D lab has a Quality Management System in place that allows us to produce these devices under the current MDR, including fully supported dossier building. This way, we can deliver patient-specific solutions for specific (spine surgery) problems that previously could not be treated. Another major benefit of having an in-house design process is the intensive interaction between the 3D lab and the surgeons. During the design of these devices, we regularly have multidisciplinary meetings, sometimes multiple times per week. Visual models can be printed in-house, quickly obtaining a better insight into patient anatomy. This allows us to accelerate the iterative steps in the design process and shorten the lead time from scan to surgery [35,36].

This 3D technology can help hospitals with perioperative models for surgical training and patient awareness [37,38]. Furthermore, 3D technology helps physicians to increase their accuracy and allow for options that were previously not possible using conventional manufacturing methods [39,40]. However, there are also limitations for the point-of-care production of in-house-developed devices. Physicians should only opt for patient-specific medical devices when a conventional commercially available treatment is not available or would not have the desired outcome, as commercial implants are considered safer due to even stricter registration conditions. Furthermore, the treating physician should weigh the added value of a personalized implant against the costs, which are especially high as a result of the laboriousness of the process. However, the labor decreases with established pathways and the costs for production decrease as the market matures.

Another limitation of implementing 3D-printed patient-specific implants into regular clinical care is the lack of reimbursement of costs by the insurance companies. In many countries, the use of patient-specific medical devices, as described in this paper, are not part of standard care [41]. One of the reasons for this is the lack of evidence, which is hard to establish for exceptional and very diverse cases. However, as the custom-made devices market matures, the advantages will become evident, followed by reimbursement solutions [41].

## 8. Conclusions

In this paper, we showed two successfully implemented 3D-printed patient-specific device pathways that were developed in collaboration with a point-of-care 3D lab in an academic hospital, leading to short lead times for products that comply with current (inter)national regulations. The establishment of such a 3D lab for in-house development demonstrated great value for tertiary referral hospitals that regularly see exceptional cases, which demand fast and exceptional solutions.

## Supplementary Materials

The following supporting information can be downloaded at: https://www.mdpi.com/article/10.3390/jpm12030395/s1, Figure S1: The cervical pedicle click-on guides consist of patient specific designed drill trajectories (**A**) and lamina hooks (**B**). Thereafter an interconnecting bridge (**C**) and a registration pin-hole system (**D**) are designed. If needed the components can be disassembled from each other using the disassembly hole (**E**). (**F**) Shows the locking box that keeps the cranial and caudal component in place. The inserted metallic drill guide is visualized in (**G**), Figure S2: Individual components of the click-on spine guide. From top to bottom: Cranial component, metallic drill tubes, caudal component, fixation box. First step in assembly: The cranial and caudal part are positioned on to each other. Second step in assembly: The drill tubes are set in position. Third step in assembly: The fixation box is positioned around the cranial and caudal components to create a shape confinement, Figure S3: 3D-Printed click-on drill guide system with metallic inserter, Figure S4: In red the dockingparts are visualized. They contain an porous bone-implant interface. In green the bridging part of the implant is annotated. In yellow the screw directions are highlichted. The directions of the screws are being safeguarded by the use of an intraoperative drill-guide.
