# Supplementary material for: Vital Role of In-House 3D Lab to Create Unprecedented Solutions for Challenges in Spinal Surgery, Practical Guidelines and Clinical Case Series
Source: J Pers Med. 2022 Mar 4;12(3):395. doi: 10.3390/jpm12030395 (PMC8951204; doi:10.3390/jpm12030395)
Supplement: Supplementary file 1 [file jpm-12-00395-s001.zip › jpm-1612982-supplementary.pdf]

Supplementary Data 1:

Detailed design click-on spine guides:

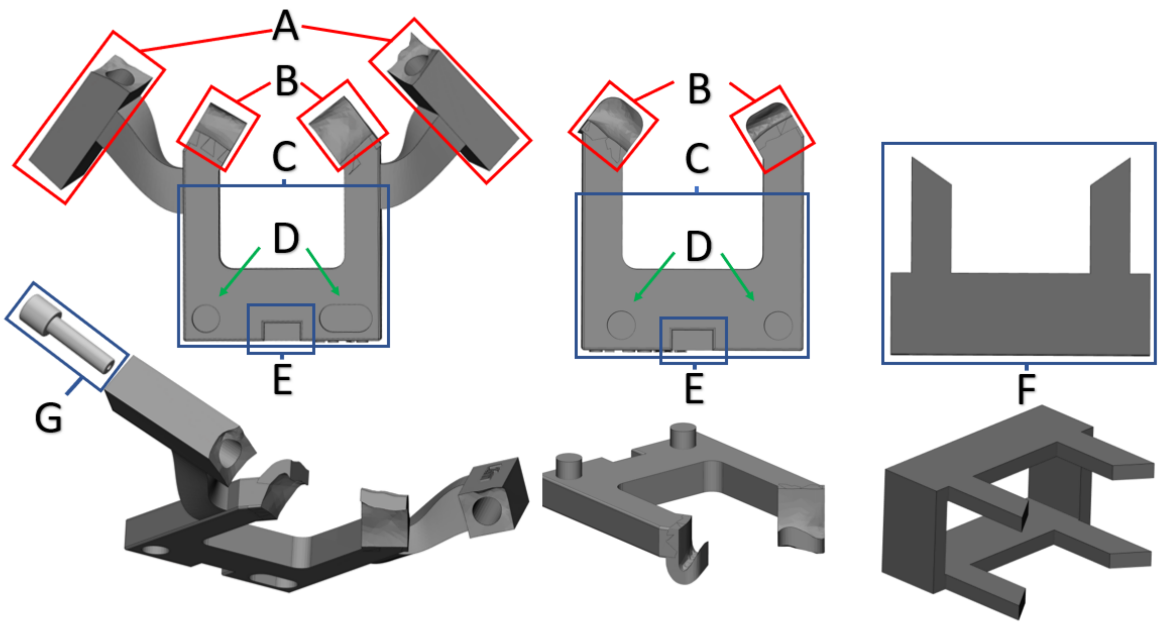

Figure S1. The cervical pedicle click-on guides consist of patient specific designed drill trajectories (A) and lamina hooks (B). Thereafter an interconnecting bridge (C) and a registration pin-hole system (D) are designed. If needed the components can be disassembled from each other using the disassembly hole (E). (F) Shows the locking box that keeps the cranial and caudal component in place. The inserted metallic drill guide is visualized in (G)

Drill guides (A)

The drill guides (Fig. S1A) are a part of the cranial component. A beam is used to connect the drill guides with the bridge (C). It is placed as closely as possible near the Cranial Lamina Hooks. This grants the stability of the firm grip of the lamina hooks to the drill guides. The drill guides consist of a hollow cylinder to allow a 3.0mm stainless-steel tube with an inner diameter of 1.28mm, specified for a 1.25mm guide wire (Fig. S1G & S2).

Lamina hooks (B) and bridge (C)

The lamina hooks (Fig. S1B) in the cervical pedicle guide, are used to grab around the cranial and caudal edges of the laminae. They prevent translation and rotation of the cervical pedicle guide in all directions. Each pair of cranial or caudal lamina hooks is interconnected by a bridge (Fig. S1C) that follows the posterior section of the spinous process.

Registration system (D)

The registration system is used to accurately guide and hold the two components in the desired position. It consists of one slotted and one round hole in the cranial component and two round registration pins in the caudal component. The round hole fixates a registration pin to stabilize the guide in the x- and y-direction and the slotted hole stabilizes the guide for rotation.

Disassembly mechanism (E)

The purpose of the disassembly cavity (E) is to allow easy separation of the two components, which is crucial during the surgical procedure with limited space and sight. It is a rectangular shaped opening that can fit a surgical tweezer or rasp. Twisting the tool within the cavity will disassemble the cervical pedicle guide.

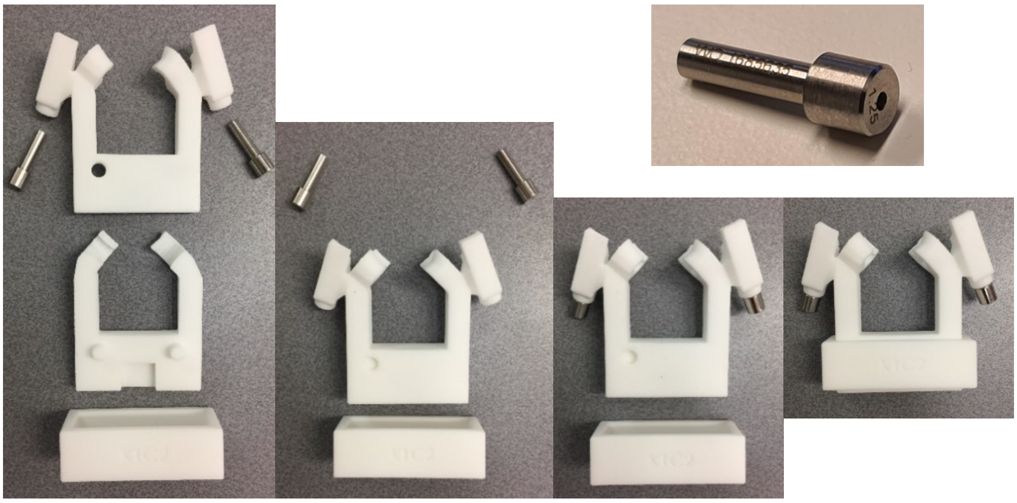

Figure S2
Individual components of the click-on spine guide. From top to bottom: Cranial component, metallic drill tubes, caudal component, fixation box.
First step in assembly: The cranial and caudal part are positioned on to each other.
Second step in assembly: The drill tubes are set in position.
Third step in assembly: The fixation box is positioned around the cranial and caudal components to create a shape confinement.

Fixation or confinement box (F)

The guide can be positioned by first hooking the bottom part around the caudal side of the laminar arch, then clamping the top part on the cranial side, and as a last step locking the mated parts using the fixation box.

The fixation box is a straight-forward rectangular box that slides over the cranial and caudal component after the cranial and caudal parts are assembled. The shape of the fixation box can vary to create extra pressure on the components, this depends on the patient's anatomy. To disassemble the cranial and caudal part of the guide after drilling the fixation box needs to be removed (Fig. S2)

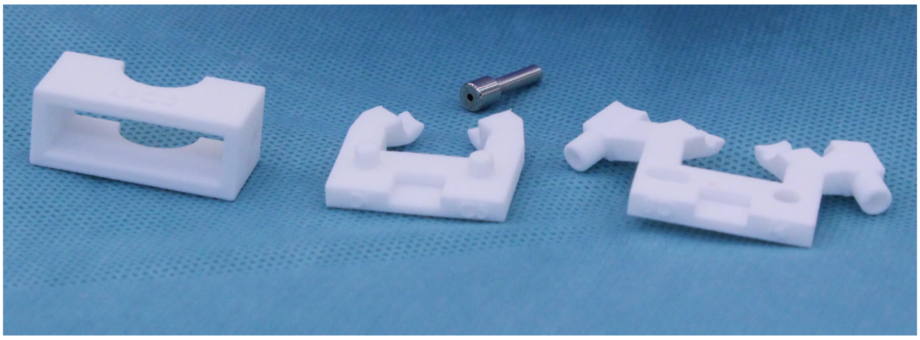

Figure S3. 3D-Printed click-on drill guide system with metallic inserter.

__Supplementary Data 2:__

__Detailed design of spinal column prosthesis:__

The aim of the prosthesis is to bypass the anterior spinal column and take over the support role. Designing requires a different approach per patient depending on the spinal segment that needs to be supported but the general steps are similar for all. To prevent shear stresses on the bone-implant interface it is important that the spinal implant is in line with the biomechanical axis of the spine.

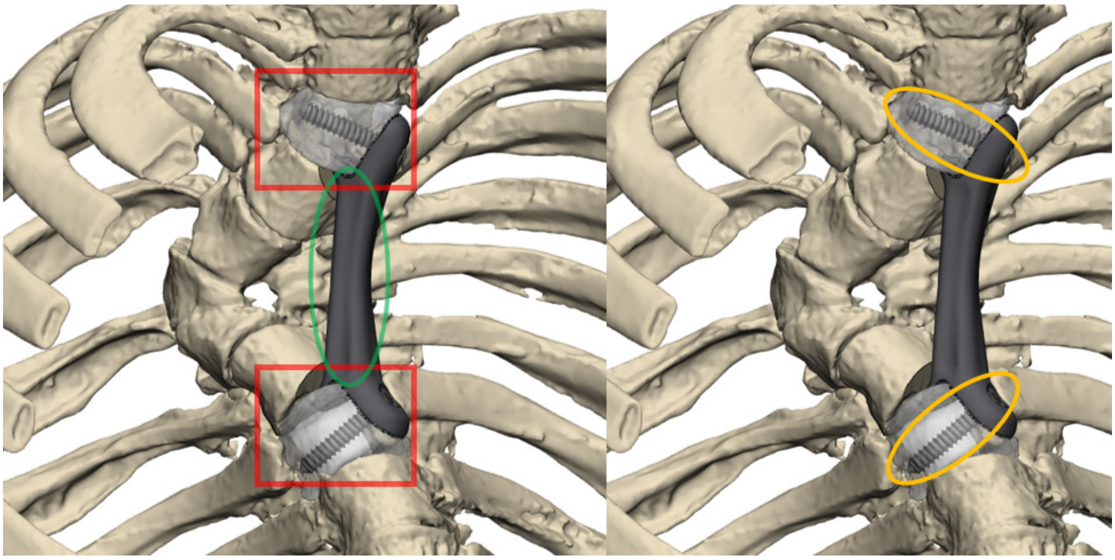

Figure S4. In red the dockingparts are visualized. They contain an porous bone-implant interface. In green the bridging part of the implant is annotated. In yellow the screw directions are highlichted. The directions of the screws are being safeguarded by the use of an intraoperative drill-guide.

Docking parts

These parts act like a buttress and support the lower vertebral endplate of the most proximal vertebra and upper endplate of the distal vertebra. The buttress is docked in the intervertebral disc space and fixated to the vertebra with a preplanned 6.5mm cancellous screw (Fig. S4) To facilitate integration with bone the interface with the bone is made of a 1 mm layer of trabecular metal (70% porosity, 500 μm pore size diamond cell structure). The buttress docking needs some resection of the by-passed vertebra. To guide this a trial docking implant is used (Fig. S4). To guide screw positioning a 3D-printed polymer guide is used.

Bridging part

The bridging part connects the buttresses and receives all the load. The cross-sectional area of the bridging part is made as large as the anatomy allows with at least $1cm^2$.
